# Perceived space characteristics fostering friendship with place, peers, and nature in the preschool yard

**DOI:** 10.3389/fpsyg.2025.1613637

**Published:** 2025-12-01

**Authors:** Fredrika Mårtensson, Amanda Gabriel, Inês Félix, Annika Manni

**Affiliations:** 1 Department of People and Society, Swedish University of Agricultural Sciences, Alnarp, Sweden; 2 Department of Applied Educational Science, Umeå University, Umeå, Sweden

**Keywords:** outdoor environment, preschool, children’s play, education for sustainability, sense of place, place attachment, nature-based solutions, landscape architecture

## Abstract

Preschool facilities in dense urban conditions with more plain open spaces and artificial materials put children’s access to nature at risk. In the transformation towards sustainable futures, children’s place preferences can be an important guide to the planning and design of outdoor environments where they can develop bonds and friendships with place, peers, and nature. Research has documented useful features for children at a preschool yard, but less is known about how children make meaning of the space, and the role of the physical environment for their development of sense of place. This study aimed to investigate favorite places during walk-and-talks in preschool yards with children aged 3–5 years old in a Swedish municipality. Field notes, maps, and photos documented how the children use and make meaning in the preschool yard. The results are six perceived space characteristics: sandbox space, artificial dwelling space, bushy space, woody space, borderland space, and temporary space. The results are discussed in the light of bonds with place, peers, and nature as formative of children’s place-identity and burgeoning development of sense of place. It suggests that more attention is paid to the general characteristics of children’s outdoor spaces as they are perceived by children, in addition to basic requirements for size and functionality.

## Introduction

In “Childhood nature connection and constructive hope,” Chawla and Gould (2020) provided an overview of how early connections with nature shape emotional and behavioral foundations for sustainable everyday practice. There is a window of opportunity for children to develop a sense of connectedness with nature during their preschool years, when body and place are more intertwined (Annerbäck et al., 2024). Outdoor environments with the presence of nature contribute to the children’s healthy development (Mygind et al., 2019; Söderström et al., 2013) and are spaces where children can develop bonds to some places (Chawla et al., 1992) and friendships with a larger array of places (Chatterjee, 2005). These are all places potentially formative for their development of “self” and place-identity resulting in certain place-preferences (Proshansky et al., 1983) associated with certain “scripts” for activities and “schemata” for places at the cognitive level (Bonnes and Lee, 2003), which are more or less compatible with sustainable ways of living (Prévot et al., 2018). Further, children’s contacts, explorations, and playful interactions with nature at a young age have implications for their environmental understanding (Beery and Jørgensen, 2018), preparing them to take on environmental stewardship (Prévot et al., 2018; Wells and Leksies, 2006).

An overall densification in the planning of new developments has changed the game for children’s outdoor stay (Mårtensson et al., 2017), with less space left between buildings for children’s outdoor stay and play (Jansson et al., 2022). Many new preschool facilities are multi-story facilities surrounded by open surfaces of pavement and artificial material (Manni et al., 2024). Urbanization has led to an overall decline in biodiverse local ecosystems (IPBES, 2019), and ecologists talk about an “extinction of experience” where some children do not get the chance to encounter wild nature, or living organisms at all, in their daily lives (Gaston et al., 2020). Many children depend on local ecosystems around their school for encounters with nature in everyday life, experiences supportive of their education in sustainability (UNESCO, 2020) and stipulated for them by the Swedish preschool curriculum (National Agency of Education, 2018).

Asking children to show us their favorite places while sharing their narratives about these places is a way to explore what and how “space” is turned into a precious “place,” as Tuan (1978) formulated the pathway of becoming at home in a setting. The development of a “sense of place” is a multidimensional endeavor characterized by embodied engagement with place, having physical, social, emotional, as well as cultural connotations (Gillic et al., 2024). Children, and especially the very young, make meaning through their direct transactions with the physical environment of distinct spaces [see for example, Harker (2005) and Kraftl (2013)]. Jørgensen (2017) points out that the word “sense” contains an ambiguity that reflects how intertwined the “sensing” of place through the senses, is with symbolic meaning-making in processes where the relationship with place is re-negotiated, and redefined continuously (Raymond et al., 2021).

The process of developing bonds to a place, and starting to feel “at home,” is the result of dynamic transactions between self and surroundings. Children’s embodied and emplaced approach to their physical surroundings form transactions with place loaded with emotions (Harker, 2005; Bartos, 2013; Hackett, 2016). In children’s approach to their surroundings during outdoor stay, there is a tension between their appetite for exploration and their need to affiliate with places (and people) where they feel safe and secure (Chawla et al., 1992; Kahn and Kellert, 2002). In his thesis (Var hör människan hemma?), the architect Bobo Hjort (1983) vividly describes the existential dimension in a child’s detection of place as ways of “being in the world” (in Swedish “varandemöjlighet”). He exemplifies with the child exploring different features of a playground, now and then returning to the ultimately trustful and secure relationship with his dad, staying put by a park bench. Further, the appearance, smells, sounds, and light of this place become associated with distinct feelings through repeated use (Hjort, 1983, p. 144). Stern (2010) describes how relationships with people and places are accompanied by short-lived “vitality affects” accompanying situations as “strands of music,” which impregnate every event (Sommer, 2012) and the places associated with them (Mårtensson, 2004).

Children’s embodied approach to biodiverse outdoor settings concurrently triggers their mobility and imagination, resulting in a play flow of transactions with peers and place that evolves in a chameleon way (Mårtensson, 2004; Sallnäs Pysander et al., 2024). Their strong attachment to place is also associated with attention to detail (Ergler et al., 2021; Chatterjee, 2005), tuning into nature (Prins et al., 2022), and bonding with animals (Byström et al., 2019). Further, when children feel safe and secure and experience some freedom at the same time, they can experience “group-glee,” which are particularly joyful interactions with peers and place (Lökken, 2009). Such episodes of joyful interaction have been documented during children’s play in proximity to nature in preschool yards, for example, when they lie close together in the shrubbery, eating currants while laughing (Mårtensson, 2004). Other peak experiences typical of the preschool yard are sequences of thrill and excitement as they climb heights, run fast, or hide from the gaze of adults (Sandseter, 2009a, 2009b).

Outdoor stay in preschool is also embedded in the socio-cultural practice of education (Klaar and Öhman, 2014; Engdahl, 2014) with outdoor spaces taking on symbolic significance as “classroom,” “home base,” or “fairyland” (Änggård, 2016). The presence of nature adds zest to children’s imaginations (Jørgensen, 2016). It is even argued that the outdoor environment lends itself to a freer use, beyond the stricter norms for children’s behavior that tend to prevail indoors (Annerbäck et al., 2024).

There are many commonalities in place preferences documented across different populations of preschool children (Woolley and Lowe, 2013; Johansson et al., 2020). The “affordance” concept from ecological psychology (Gibson and Pick, 2023) has been applied to uncover the many functional properties associated with nature-based playgrounds (Lerstrup and Konijnendijk van den Bosch, 2017). Niklasson and Sandberg (2010) identified climbable features, shelters, and moldable material, but Hagen and Skaug (2022) found children favored places containing play equipment. A study in Australia reported that open surfaces, were particularly attractive to children, besides the (often limited amount of) nature present in these preschool settings (Dyment and O’Connell, 2013).

The dynamic interplay between children and the physical environment, shaping children’s bourgeoning development of a sense of place, is embedded in the socio-cultural practices (Gibson and Pick, 2023) and the type of landscapes available (Berkhuizen 2020; Mårtensson, 2004). Common to many contemporary childhoods is the fact that the preschool yard is one of the few settings where young children get the chance to become acquainted with the wider world and its people. Sudeshna Chatterjee (2005) describes how children bond with favorite places, associated with experiences of beauty, feeling in control, freedom, and escape from social pressures, but also develop friendships with a larger network of places, important for their overall socio-emotional development.

The literature contains investigations into the useful affordances of preschool yards and qualitative investigations into preschool children’s transactions with place, peer, and nature, but we know less about the overall adequacy of the preschool settings offered to the children. Ethnographic reports with observations of how children use their bodies and senses to explore space, is at the core of research on playgrounds (Lerstrup and Konijnendijk van den Bosch, 2017), as well as research on children’s “place making” and development of a sense of place (Chawla et al., 2014), where children guide us into their everyday settings, showing and telling us about their favorite places (Cele, 2023; Korpela 1989, 1991; Korpela et al., 2002). This study is an effort to uncover how the functional properties combine with more elusive dimensions of space in children’s place-making. It is an emplaced and embodied perspective on place with dual attention to the agency of the physical environment and that of children (Annerbäck et al., 2024). The questions is what spatial characteristics are vital to children’s embodied dwelling as they develop a sense of place in the preschool outdoor environment? “The perceived sensory dimensions,” which define restorative characteristics of green space for adults (Bórques, 2025; Stoltz et al., 2024), have inspired the design of nature-based playgrounds (Beckman et al., 2022, pp. 30–31). “The “perceived space characteristics” should add to this by offering a “smorgasbord” of features supportive to the very youngest children.

This study aims to investigate children’s place preferences in preschool outdoor environments to determine which type of spaces are important to the very youngest children’s burgeoning sense of place. How do the children approach, use, and make meaning of the physical environment during their interactions with place and peers? What are the characteristics of space that children prefer?

## Materials and methods

### Research design

This research on the implications of contemporary design for children and preschool practice involved researchers in environmental psychology and applied education. The data collection was conducted in fall 2022 in one of the larger communities in northern Sweden. It investigates children’s approach to preferred locations and favorite places in their preschool outdoor environment through guided walk and talks. The study situates the outdoor environment in a socio-ecological context (Mårtensson et al., 2017), where the structure of a setting, as well as children’s image of it and their actions, are studied concurrently (van den Brink et al., 2017, p 15). In line with ecological psychology and the concept of “behavior setting” (Barker, 1968), it focuses on standing patterns in children’s use of the environment. Further, it adheres to an embodied perspective on children’s place experience in which descriptions of children’s affective responses to place collapse with descriptions of attributes in the physical environment (Annerbäck et al., 2024). This combined approach paves the way for typologies of the outdoor environment, which can predict both the type of activity and its valence (Mårtensson, 2004; Sallnäs Pysander et al., 2024; Stoltz et al., 2024), in this case a set of Perceived Space Characteristics.

### Sample of preschools and children

The sample of preschools was the result of an iterative process set up to select a variety of the most common designs available in the full stock of facilities in the municipality. The children involved in the project were 3–5 years old and came from a stratified sample of 18 preschools, with eight facilities built during 1952–1989 and 10 during 2013–2021. The sample covered facilities representative of preschools in the municipality in terms of their overall layout with an average size of 3,241 square meters (range: 957–5,729). The median number of children enrolled per preschool was 76 (range: 35–136). All facilities featured a mix of play equipment and green areas, but the newer facilities had more plain open spaces and less greenery in proximity to the buildings.

### The walks and talks

In total, 56 walks and talks were carried out with groups of 2 to 5 children, summing up to 150 children participating in the study. A walk would take about 25 min, but some took only 10 min, while others took up to 40 min. During the walk and talks (Cele, 2023; Ergler et al., 2021; Änggård, 2016), the researchers worked in pairs with groups of children, inviting them to show and describe the places they favored and used most. The researchers introduced the project and asked the children to guide them into their outdoor environment. Questions asked during the walks were “Where do you usually play?” and “What do you like to do there?” Once in a space, probes like these were used: “How do you use this place?” “What do you use here?” and “What makes this place fun/interesting?” Children were encouraged to add to each other’s narratives, and efforts were made to include all the children in the conversation.

The children’s choices of spaces, narratives, and actions were documented in multiple ways. The spaces were marked on maps, and their features and content were photographed at the children’s eye level. Audio recordings documented the children’s narratives with recollections of how they use and make meaning of the outdoor space. Field notes documented the affordances of spaces and of children’s transactions with peers and place.

### Data analysis

In total, across all preschools, 180 places of children’s choice were documented in maps, field notes, photographs, and audio recordings. The dataset contained 5–15 pages for each preschool. Furthermore, there were notes on method, procedure, and early interpretations of data. After familiarization with all the material, a thematic analysis (Braun and Clarke, 2024) with both inductive and deductive elements was undertaken. In the first round, all the places were sorted into the following tangible spatial characteristics: “natural,” “artificial,” “built,” “seasonal,” “artificial loose material,” and “natural loose materials.” A calculation of the total number and frequency of space characteristics in the material tells us how often children showed us a certain type of space. In a second round, theory on the children’s embodied transactions with an outdoor space [for example, Annerbäck et al. (2024)] informed the analysis to uncover how children made meaning of the preschool outdoor environment (Änggård, 2016; Jørgensen, 2016). The dynamic interplay among different parts of the preschool yard influencing children’s use and meaning making was also considered (Mårtensson, 2004, 2013; Sandseter et al., 2022). The “natural space” category was differentiated into three different space characteristics: “woody space,” “bushy space,” and” temporary space.” “Artificial” places with extended functions into the social realm became the space characteristics “sandbox space” and the “artificial dwelling space.” Finally, the “borderland space” category contains spaces that account for children’s attention towards the surroundings of the yard. In a third round of validation involving additional researchers on the team, the data was scrutinized for nuances, similarities, and disparities. The results present descriptive summaries of the different space characteristics, with quotes fetched from field notes and children’s telling, illustrating their approach to places in each category.

### Limitations

Data from all preschools were collected during the same period, which is an advantage when it comes to conditions (such as weather) being similar but also means that several researchers were involved in collecting the data. One team consisted of two researchers, and the other team consisted of one researcher and an experienced outdoor educationalist. The initial categorization of texts and photos was made in relation to an agreed-upon general structure, but a focus on photos in some material and a more text-based approach in others required some adaptation of the strategy during interpretation. Photos illustrating the typical attributes for each dimension are available but not published.

Some children had consent from parents but did not join the walks anyway, ultimately. The pedagogues, who helped organize the walks and talks, introduced an element of self-selection in this phase, which could introduce bias to the results, as they negotiated with the children about their involvement and adapted it to the practical circumstances of the preschool day. Furthermore, some children were less talkative than other children, which meant that the narratives of some children got more well-documented than others. In summary, the generalizability of the perceived space characteristics to all categories of children in the investigated preschools and beyond has certain limitations. The results do not aspire to cover the full range of perceived space characteristics available to children in the investigated settings. An investigation of children’s dynamic interplay with preschool settings in other regions, with other types of landscapes, can add to the Perceived Space Characteristics suggested here.

### Ethical considerations

This study followed the ethical guidelines from the authorities (Vetenskapsrådet, 2017). Before the visits, the preschools informed parents about the study in writing and obtained consent for their children participating. On our arrival, the children were informed about the project and asked if they wanted to show us around while we recorded and took notes. We tried to be attentive to any signs of children being hesitant to participate. One child withdrew from participation during the session and some of them took company of a friend or a teacher during the walk. Data was gathered, organized, and stored in accordance with recommendations for data safety and confidentiality. Since the focus was on general associations between children and space characteristics, any documentation of more personal information about the children was avoided in all steps.

## Results

The children guided us to different places of their own choice in the outdoor environment of their preschool, while we attended to their use and expressions as they made meaning through interacting with the place and peers with their bodies and senses, listening to their narratives about these places.

In 86 instances, they showed us places dominated by the natural features, and in 94 instances, they showed us places where an artificial feature was the centerpiece. The Perceived Space Characteristics recurrently appearing in the material as supportive of the children’s appropriation of space were the “sandbox space,” “bushy space,” “woody space,” “artificial dwelling space,” “temporary space,” and the “borderland space.” Of these, 18 places belonged to the sandbox space category, 37 to the artificial dwelling space category, 43 to the woody space category, and 33 places to the bushy space category. They also showed us 12 temporary spaces and eight borderland spaces. The distribution of space categories was similar for newer and older preschool facilities, except for the bushy spaces being more common in newer preschools (24 versus 9) and the woody spaces being more common in older preschools (29 versus 14).

The sandbox spaces and artificial dwelling spaces often served as meeting points in the yard for socializing with peers while carrying out activities associated with their functional properties. When they showed us places in ‘bushy’ and ‘woody’ spaces, they would often engage us in their explorations, pointing out their different features along the way. Some temporary spaces were carefully pointed out, while others they stumbled over during the walk, for example some rain puddles. The borderland spaces were significant as outposts towards home and family life, but also adventure and excitement related to ongoing events or memories from excursions. Presented below are elaborations on children’s approaches to places across the different categories, potentially supportive to young children’s place-making in an outdoor environment.

### Sandbox space

The children often pointed out the different boxes with sand during the walks. They moved sand around the yard, bringing it from one place to another or slashing a patch of wet sand onto some construction while passing. The regular sandbox, with its soft sand, was used for all sorts of activities, and the same was true fractions of sand that served as protection under equipment. Temperature and moisture affected how the sand was used and how it was experienced. The sandbox space was also important as a social meeting point. Some yards, had “the smaller children’s sandbox” and “the larger children’s sandbox.”

Some boxes allowed digging deep down in the making of elaborate and extensive constructions. They described having some fantastical goal in sight, with “small houses along roads,” “a construction site,” “a kingdom with its castle,” or “roads for toy cars.” When elaborating on trenches across a miniature landscape, their play would evolve more organically. They also demonstrated how they used the border structures of boxes for balancing and building. One girl particularly said the sandbox was an “important place” for her. She demonstrated her digging techniques to us and pointed out the usefulness of a “table to tinker with.” She filled a small truck with sand, and shaped a landscape.

A group of boys playing in a sandbox with a canal system had access to running water, buckets, and spades. They explained how they “work hard” in their “mud factory” and demonstrated how mixtures of sand and water go through various steps on the way to becoming mud, with the children taking responsibility for different tasks.

Two girls described using one sandbox for more extensive projects, where they could spend time without other children interfering. It was placed in proximity to the building. They had reached the bottom of the box while digging a “swimming pool.” They instructed a boy to fill the hole with water and eagerly invited a teacher to jump into the water. After counting to three, the teacher jumped into it with the children laughing excitedly.

### Bushy space

The children frequently invited us to join them on their expeditions into these areas, showing us their different features as they moved along. They showed us how shrubs and hedges create hidden routes which they used to move from one part of the yard to another. In bushy spaces with a hidden interior, they opened up the dense foliage by moving the branches approaching us along the routes. They pointed out “gates” and “tunnels” leading into rooms in the thicket. They showed us leaves, branches, left toys and stones, and talked about different uses and experiences associated with these features.

The overall complexity of the organic material of bushy spaces seemed to fascinate the children, and they described how the dense foliage concealed them from adults and other children. Some bushy spaces the children named:

“This tree is the umbrella tree, so if it rains you can stay under here.

Whisperingly, they also told us about their “Secret jungle”:

‘That’s the Secret jungle,’ they said, as they moved toward a dense cluster of bushes. They pointed out its different “entrances.” It is quiet except for the rustling of leaves and the children’s soft voices. Suddenly, they all stop, mouths slightly open, pointing at some current bushes, saying: Here are currants! No currents...They slowly moved into the thicket, using their hands and knees to navigate, and pointing out rooms even deeper into the undergrowth, touching leaves, branches, and stones along the way. Some of the children disappeared into the foliage.

Exploration was in focus in many bushy spaces with their many elements, fascinating the children. Often they carried sticks. They talked about spiders and insects and pretended to be scientists studying them. They instructed us not to approach a particular tree housing ants. The berries of bushes and blueberry stand made up attractive destinations. They discussed whether red or black currants tasted the best and offered us to taste. With no currents left in some spaces (an autumn day), their recollections of berries would still add positive connotation to a space.

Children referred to playing “a lot” in bushy places. Toys were left, placed, and retrieved from these spaces. Different sections of the area were associated with distinct sensory experiences, which they pointed out and associated with distinct activities. They recalled playing “hide and seek,” “monkeys,” “family,” and roles of secrecy and surveillance where they moved in confined areas, as “spies,” “detectives,” or some superhero. One narrative was about scaring all the animals out of the bushes so that the children could examine them.

Some bushy spaces contained small trees. They showed us how they could bend a whole tree down to the ground, stand on it and make it bounce back, or rock the stem from side to side to signal their location to other children inside the thicket.

“I like to go to trees and climb, but this tree is just straight, so you cannot climb it.”

### Woody space

In preschools with access to larger trees, the children would often guide us to areas they called “the forest” or “the woods.” These woody spaces refer to small patches of trees as well as larger areas with a canopy and forest floor beneath. The children often mentioned the various challenges associated with playing and staying in these spaces and also recalled episodes of imaginative play that they staged for us. As we approached a forest of pine and fir, they instructed us to follow them. They ‘fixed’ a ‘door’ by propping a crowbar against a tree trunk and declared:

“Researchers, we are now lost, we do not know how we will be back home! I have a special camera that shows footprints in the ground, and we can use it to find our way back to the world.”

The children listed the features of the forest that interested them, such as the uneven ground with visible roots, fallen leaves, stones and holes. They pointed out clay-like substances hidden in the bark of a tree and collections of natural materials, such as leaves, sticks, and stones. They also drew our attention to the presence of insects and birch bark with drawings made by children.

Children paid attention to trees and talked about them being “kind” and “friendly.” They would make some large solitary tree into a showcase for us. Some trees were given names as “the school birch” and “the large tree.” One girl hugged the birch and told us she would go there with some of her friends. They pointed out distinct attributes such as tall trunks, expansive canopies, and sturdy branches, used for climbing, hanging, and swinging. “I climbed all the way to the top,” said one child, also showing us how ropes in between two trunks allowed balancing and swirling around. The uneven ground added affordances. One child stepped up on a stone and exclaimed:

“Our preschool yard is super fun, because you can stand on stones in this way.”

Woody spaces were also spaces associated with episodes of pretend play. They told us about playing “castles,” “ships,” and “jungle.” They also associated woody spaces with “hide and seek” and showed us how they would hide behind large trunks and use the shadows of trees in their games.

In one piece of larger woody spaces, the children claimed they had “played there a 1,000 times.” Here, they showed us around, to one place with a large fir tree, to another place with a multi-stemmed leafy rowan plant, and a third place with a mix of trees, stones, and left timber. In another yard, with a piece of pine forest, they had a play-boat where they would “eat and get dirty” and a bonfire for “bath and sauna”. Skipping through the forest between these two places, they would do “horse jumping competition,” think about snakes in holes, and and make a “frog -stone” jump.

### Borderland space

There was often a “borderland space” in the fringe of the yard that the children visited. They directed our gaze to people and events outside and to features of the surroundings they found interesting. They made recollections of using these spaces as passages when going on an excursion, visiting an adjacent schoolyard, and home. One child said,

“I would like to cross the boundary, because then I could go to my favorite place, home, which is beyond the boundary.”

One preschool had important borderland space in many directions around the yard: Towards open fields with lambs grazing in springtime, deep forest, as well as two playgrounds siblings could appear. In another preschool, the children showed us a fenced corner where they could watch trucks deliver food.

Heights are another type of borderland space, destinations in their own right, attractive spots as move across the yard. Children can get an overview of their surroundings. Here they run and swirl around and experience the sky. Some children pretend to look through binoculars from the top.

### Artificial dwelling space

The artificial dwelling space is a site where children can distinguish themselves from the surroundings by staying in a delimited space with some distinct functional properties. This type of space form. form a nexus in the children’s social life in the yard. They are also hubs for withdrawel and restoration, wheter alone or with friends. When close to the buildings, they tend to become arenas for social gathering and interaction. Children can gain a distance or a distinct position in a swing or a slide, while other structures with more solid borders offer shelter and enclosure. There are artificial dwelling spaces containing climbing structures, slides, swings, huts, and spring rockers offering diverse affordances for climbing, sliding, and jumping. Their well-defined functional properties, which invite distinct activities, can help children accommodate and structure their interactions. Children described how they made room for each other by adjusting their bodies and their activity, and waiting for their turn.

A common feature of many artificial dwelling spaces was the opportunity for a child or a smaller group of to establish themselves in the space, while joyfully engaged while joyfully engaged in some bodily activity. Frequently, they would show us the affordances of a space by carrying out a sequence of activity while they moved around. Some spaces they approached alone, other features in pairs, and even others in groups. Some features, like buddy swings and spring rockers, adapted to a limited number of children, rendered stories about how they had to line up to get a chance to use it. Two girls laughingly sled down a strip of plastic running down a hill across the wet lawn, right after the morning rain, calling out, “Much better slide with this! Look!”

“There are huts here!” they exclaimed and quickly began exploring the structures and collecting “treasures” such as mushrooms, leaves, and stones. Some huts together formed a small marketplace or village. They told us about hut areas affording solitude and restoration, as well as opportunities for socialising and exploration. They explored a piece of wood in a structure and exclaimed, “There is sand, feel! They climbed the interior of a hut and said they would “climb higher than the moon.” The huts were also associated with playing “house,” “family,” “store,” factory” and carrying out a distinct activity like “watching television” and “having dinner”. Several of their stories elaborated on both real and imagined relationships between the place and its people. One boy told us it was the “best day of his life” when he sold “hot dogs” and children lined up in a queue that stretched outside the yard.

### Temporary space

Children often drew our attention to features in the yard that changed over time, temporary spaces that reflect the ongoing changes of any outdoor space exposed to weather. The children showed us things like water puddles, piles of leaves, and patches of dirt, sand, mud, and gravel. They would pick it up and demonstrate how it could be used, mixed, or shaped. Water was a recurring theme among the temporary spaces, with puddles making up miniature lakes for play.

The children would in different ways confirm to us that they acknowledged the temporary character of these spaces. One child carefully drew lines with a rake in a soccer field of gravel while telling us these patterns would only be temporary. Another child said about a puddle:

“The rain made a big puddle! We can float things in it. But it will disappear.”

They encouraged us to have tactile interaction with the elements. One child silently guided us to a place where water was dripping from the roof drainage, placing the hand of a researcher under it. Another child pointed out how the temperature of the surface of an electrical cabinet changed with the weather:

Look at this metal box, feel it with your face. It is so cold, can you feel it?”

The visit to one temporary space would often lead to the exploration of another temporary space in another part of the yard, to a place expected to have more, or other types of affordances:

“Two children picked up rakes from the storage area and began to gather scattered leaves on the ground. Then they ran up a hill and tried to get a toy car to move downwards in the running water. ‘It’s flooding over the car!’ They talked about picking flowers in the woods to add to the site. When the toy car hit a tree trunk, a teacher told them to stop.”

They also showed awareness of seasonal influence, particularly of things growing. They showed us a pumpkin. They talked about bushes that had had berries, trees that were bearing fruit, but also flowers that had never blossomed. Bushes that once had provided an abundance of berries became spaces of anticipation when the berries were gone:

“Last time there were blueberries here, but now they are gone. Maybe we will have to wait for them to grow back.”

They also considered seasonal variation influencing the overall use of the yard. The benefits of winter and the benefits of summer:

“Maybe next winter, the ice will come back and we can slide again!”

“In summer, we can hide here, but in winter, there are no leaves, so it’s not a good hiding place anymore.”

## Discussion

Many children rely on outdoor spaces at preschool and school to experience nature and enjoy self-directed outdoor activities with their peers (Annerbäck et al., 2024). Sutton-Smith (1997) even talked about the school yard as an ongoing festival for children with its intense social life. The departure point of this study was the assumption that some types of spaces are more useful than others, as preschool children appropriate and try to make meaning of space in their bourgeoning development of a sense of place. In this empirical interrogation into a large sample of preschool yards in a Swedish municipality, we identified six different “Perceived Space Characteristics” in the preschool outdoor environment supportive of children’s development of bonds to places (Chawla et al., 1992).

The interrogation focused on children´s favourite places (Korpela 1989; 1991) but also incuded their lighter “friendships” with place, across a larger number of settings (Chatterjee, 2005) which add meaning to outdoor stay in preschool and become important part in their life long development of sense of place. took departure in the insight that young children’s relationships to place are the result of their embodied use of functional properties immersed in affective and socio-emotional content related to the very dynamic interactions with peers and place in a preschool setting. Given that, the functional properities already are well documented (Lerstrup and Konijnendijk van den Bosch, 2017; Mårtensson, 2004), the intention here was to include in the analysis a layer of affective and socio-emotional transactions involved in young children’s place-making. The strategy of asking for place where they “like to stay and play” had the intention to guide us to places important to the children, grounded in embodied insights of how the spaces outdoors had nourished them over time, during everday events at preschool.

The ‘sandbox spaces’ and the ‘artefact dwelling space’ are social nexuses at the yard, equipped with functional properties that serve as invitations to children’s play and exploration. These spaces satisfy children’s desire to engage directly with their surroundings, allowing them to explore their affordances. The transition from a stage of exploration to the appropriation of space, can be quicker in these areas than in spaces dominated by natural features where forms are more ambiguous and open-ended (Waern et al., 2025). Its distinct borders and structures can shelter from the buzz of events in the yard. Sometimes a small number of seats helps to limit the access of other children. An element of territoriality can make children “own” a space by arriving there first, or by being older and more persuasive. This ownership of a place in the preschool yard, for one child, a pair, or a group, can convey a comforting insight of having “somewhere to be and something to do” during outdoor stay.

It is important to note that not all sandboxes or play equipment in a preschool yard belong to the Perceived Space Characteristic; only those where children dwell. Typically, a sandbox space has a very low threshold for involvement. A hut structure, also easily lends itself to play, triggering ideas of safety and shelter (Sobel, 1993) associated with scripts and schemas (Bonnes and Lee, 2003) for “house,” “family,” etc. It is hard to evaluate the status of spaces from single visits since children use some of their features for only a short while. They can take a couple of rounds on a swing or stop by the huts to explore, on the way to another destination. However, the integration of equipment and sandboxes with vegetation tend to increase their usefulness substantially (Jansson et al., 2022) making it more likely the space becomes a social nexus at the yard. One example was the adventourous pretend play evolving in the pine forst with a play boat.

The children frequently showed us “bushy spaces” and “woody spaces” during the walks. Similar to the two types of spaces was the way children took us with them on an “expedition” to share their experience and perceptions of these spaces, rather than just pointing them out and telling us about them. They would direct our attention towards details and display through their actions how they interact with the place and peers in this type of space. Some play episodes staged, seemed to be recollections of earlier play episodes. The hidden interiors of the vegetation contained sub-settings which they referred to as places for retreat, where they would withdraw to restore and experience a sense of wonder. In another preschool, they staged for us a drama related to not having the possibility to visit “The forest border,” an area fenced off and only opened occasionally. In the preschool with the largest piece of forest in the sample, the children referred to it as “The deep forest.” The implications of children having access to land with forest could not be underestimated, as we know that it contributes to making children’s play more active, versatile, and varied (Sallnäs Pysander et al., 2024) and has direct implications for the health of preschool children (Puhakka et al., 2019; Söderström et al., 2013).

We applied a situated perspective on the preschool outdoor environment, as a setting where “the landscape is playing with the children” (Mårtensson, 2004), with its functional properties embedded in the social dynamics of peers and pedagogues and the variability of season and weather. The edges of a preschool yard also contain inherently interesting spaces due to their very location, making up “borderland space” where siblings may appear, goods enter, the gate opens for an excursion, and parents arrive. In other borderland spaces, they watch the sky. Finally, we identified a bundle of “temporary spaces” which due to growth, season and weather contain a dimension of impermanence (Ergler et al., 2021). Their narratives contain many examples of excited interaction with this temporality of the yard, with water puddles and berries being treasures, with information on how “things work.” This dimension This dynamic perspective, integral to all nature, becomes a centerpiece of children’s attention. One study showed, that the presence of rainwater can increase the overall physical activity of children at a preschool yard (Boldemann et al., 2006).

The Perceived Space Characteristics documented during walks with the children should give us an idea of how children make sense of a preschool outdoor environment and feel at home in a preschool yard. It gives us a hint of how different types of spaces becomes part of the dynamic interplay between children, space and peers, but more importantly for this study, what this implies for their appropriation and development of a sense of place. Some of the sandpits, playhouses, and other artificial dwelling spaces seemed to be vital parts of children’s play with shared ideas and scripts for what they could do and experience in the different settings. Other types of equipment gain little attention and might be of less use for children’s place-making in a preschool yard.

The Bushy, woody and borderland space were places they were markedly proud and exhilarated to show us, staging for us their shared experiences of their different attributes. They exemplified with narratives from imaginative play and gave places names such as “the forest edge” and “the rainy bush” (Jørgensen, 2017). Many of these spaces were in the fringe of the yard, however, some children had “dens” in a single bush or piece of shrubbery close to the buildings. It highlights the importance of vegetation in areas close to buildings to make the yard inclusive, also to the (younger) children seeking shelter and shade in proximity to the pedagogues.

Children can use the outdoor environment for rest and recreation as they explore their preschool yard. There are documented health benefits of children having access to green and spacious outdoor environments, promoting vigorous and versatile play in interaction with peers and nature (Söderström et al., 2014). However, a development into larger preschool facilities with less space and greenery and more paved surfaces puts children’s health and development at risk. Space requirements of outdoor play settings are important (Kylin and Bodelius, 2015), but we also need ideas on how to strengthen the overall carrying capacity of the surfaces available. The idea with this study was to identify tangible characteristics of outdoor spaces capturing also their more elusive dimensions of human experience (Stoltz et al., 2024) used and favored by children in their preschool outdoor environment.

The children’s selection of places during the walks probably gives a rough indication of favorite places among preschool children children in the region. The walks, not being very long and often starting by the building, for sure influenced the selection of spaces, making it more likely that they showed us some spaces than others. Maybe they favored spectacular forest spaces to “show off” sometimes, or assumed that, as adults, we were more interested in the regular play equipment. These things we do not really know. However, the role of places being spectacular and their location at the yard, are factors influencing children’s everyday use of their yard, too, not only during a research session. In light of the high number of preschools and walks, we still think one can learn from the overall patterns observed in this sample of children’s place preferences at preschool yards. However, observation studies are better than guided walks at capturing children’s mobility and their use of paths and routes within the layout (Dyment and O’Connell, 2013; Raustorp et al., 2012). In this study, bikes and bike paths were excluded from analysis. It was based on the limited supply of bikes and earlier studies showing how bikes tend to stagger the development of more dynamic exchange and play among the children at a preschool yard (Mårtensson, 2004; Mårtensson, 2013). Some children dropped out during the walks with us researchers, and some of the children at the preschools did not participate at all in this study. In future studies, the different Perceived Space Characteristics suggested for design need to be scrutinized from the perspective of single children, considering how gender, age, and profile on capabilities add to situations and their capacity to bond and develop a sense of place in a preschool yard.

The overall presence of greenery on the preschool yards in this sample makes them representative of an older planning regime in Scandinavia when green structure was considered an important dimension of a welfare society (Pries and Qviström, 2021). There is also a new generation of more densely populated urban settings with little or no space for play outdoors in preschools (Ekman Ladru and Gustafson, 2025). This sample included larger preschool facilities, surrounded by more plain surfaces, but to some extent, they all had natural features, such as shrubbery and hilly terrain, which children used and integrated into their place-making. In the newer preschools they showed us more bushy spaces, and in the older preschools they showed us more woody spaces. It probably reflects the fact that children give priority to forest if there is any, but also that presence of bushes as part of the understory in mature forest, is limited. Some clearing in forest to let in light can create a richer understory for children’s play (Mårtensson et al., 2017) but can be hard to establish in areas with a lot of trampling. Overall, the preschool design of this municipality acknowledged the value of combining play equipment with vegetation in play settings. Children’s access to woody spaces was limited, in spite of the forest being the dominating land type for the area. Much more attention is required on how local ecosystems can be protected and developed, supportive to children’s development connection to peers, place and nature in their development of sense of place (Mårtensson et al., 2025).

When the planning and design of preschool outdoor environments give priority to artificial material over natural features, it does not mean that the children’s place preferences change. In ambitions to make natural material and features available to children, we can trust in children’s capacity to navigate in and make meaning out of their complexity, here illustrated by their confident approach to bushy and woody spaces, and their attentiveness to ephemeral aspects of temporary space. When there is forest beyond the fence of preschools, there are many reasons to make it available to the children, when not, one need to uncover what natural resources can be made avialble to children in that context. We hope that work on ´Perceived Space Characteristics´ will help to maintain and develop preschool outdoor environments that make sense to children in the everyday life, offering an outdoor space where they can feel at home with place, peers and nature in an extended network of spaces. We would argue the outdoor spaces made avialble to young children have a lasting imprint yards have a lasting imprint on children’s place preferences and overall relationship to people, place, and nature, with implications for the welfare of children, and sustainable futures of our societies.

## Conclusion

In Sweden, as in many socieities, schoolyards and preschool yards are vital for children´s possibility to outdoor stay in contact with peers, place and nature, places with impact on their health and development and capacity to environmental stewardship. Environmental stewardship. Children’s play evolves in relation to the features of the landscape, with children alone or in pairs or groups coordinating their movement across the space by taking direction towards landmarks and responding to various affordances for bodily movement, such as jumping, climbing, and hiding. This study provided an overview of the Perceived Space Characteristics that children utilize when they appropriate an outdoor space and establish a sense of place and belonging to the preschool outdoor environment. The “sandbox space” and the “artificial dwelling space” with their huts and an equipment are social nexuses with their readily available affordances to physical activity and to peers, while “bushy space,” “wood space,” “temporary space,” and “borderland space” create grounds for more elaborate play and exploration, stimulating children’s imagination and offering encounters with nature. In a space with an array of different space characterstics availble to them, children will find it easer to dwell, transforming the preschool yard into a “landscape of becoming” (Mygind et al., 2021), where they can connect with nature, peers, and their environment in ways that support their socio-emotional development. Children’s keen interest in growth, seasons, and other processes shaping their surroundings as they engage with places and peers during outdoor stays should inform the planning and design of sustainable futures.

## Data Availability

The raw data supporting the conclusions of this article will be made available by the authors, without undue reservation.
